# Risk Evaluation of Endoscopic Retrograde Cholangiopancreatography-Related Contrast Media Allergic-Like Reaction: A Single Centre Experience

**DOI:** 10.1155/2018/6296071

**Published:** 2018-02-15

**Authors:** Félix Trottier-Tellier, Laurence Harvey, Jean-Daniel Baillargeon

**Affiliations:** Gastroenterology, Department of Medicine, CHUS, Sherbrooke, QC, Canada

## Abstract

*Background and Aims. *Few cases of endoscopic retrograde cholangiopancreatography- (ERCP-) related contrast media (CM) adverse reactions have been reported in the current literature. There is a lack of standardisation in practice regarding premedication prophylaxis for at-risk patients undergoing ERCP and there are few data to guide the practitioners. Our goal is to evaluate the risk of CM adverse reaction in a group of patients with a past history of allergic-like reaction to iodine product undergoing ERCP.* Methods. *A retrospective chart review study was performed of patients who underwent ERCP at our single centre from January 2010 to December 2015.* Results. *2295 ERCPs were performed among 1766 patients. No anaphylactoid or severe adverse reaction occurred. One (0.04%) ERCP-related CM benign reaction was reported in a patient known for penicillin allergy. Among 127 ERCPs performed on patients with a prior adverse reaction to iodine, 121 procedures were done without and 6 with a premedication prophylaxis. In both groups, no ERCP-related CM reaction occurred.* Conclusions.* To our knowledge, we report the largest cohort of iodine allergic patients undergoing ERCP ever published. These results suggest that ERCP-related CM adverse reactions are very rare even among patients at risk for CM reaction.

## 1. Introduction

A few cases of ERCP-related CM adverse reactions have been reported in large cohort series [1]. The exact incidence rate of ERCP-related CM reaction is unknown but seems very low [2]. Several studies have documented that systemic absorption of CM occurs after ERCP and it has been shown that there is a rise in serum iodine concentration following ERCP, increasing concerns about possible post-ERCP systemic CM adverse reaction [3–7]. The paucity of data concerning post-ERCP CM reaction has lead clinicians to look up to radiologic literature and the risk of intravenous (IV) CM reaction in an attempt to better understand possible post-ERCP CM reaction. Systemic CM adverse reactions after IV CM injection are classified as acute (within 1 hour) or delayed (even after 10 days) [8]. The underlying mechanism is sometime chemotoxic, but the majority of cases are anaphylactoid or pseudoallergic [9]. They are mainly due to the release of histamine from basophils and mast cells which can cause diffuse vasodilation and bronchospasm [8]. CM adverse reactions are mostly mild or moderate, but when rare life-threatening reactions occur, they arise minutes after CM injection. Adverse CM reactions after IV injection are hard to predict but many large scale studies identified risk factors including a prior allergic-like reaction to CM, an allergic diathesis, and asthma [10–12]. Of these, the strongest risk factor identified is a prior allergic-like reaction to CM justifying a premedication prophylaxis before IV CM injection and raising the question: Should we use a prophylaxis for ERCP in patients known for a prior CM reaction?

There is a controversy concerning the prevention of ERCP-related CM reaction. The American Society for Gastrointestinal Endoscopy (ASGE) issued a guideline on the topic of radiographic contrast media used in ERCP and reported that there is no evidence-based standard of practice for prophylaxis against contrast reactions during performance of ERCP [2]. They conclude that prophylaxis against ERCP-related CM reaction might be considered for high risk patients based on theoretical considerations. Despite the lack of clinical evidence supporting prophylaxis for ERCP-related CM reaction, recent surveys among a small sample (*n* = 44) of American ERCP practitioners show that a majority (82%) use prophylaxis in patients with prior reactions to IV contrast medium [13]. Considering these, there seem to be a gap in current guidelines and confusion among practitioners as to whether we should use premedication prophylaxis for iodine allergic patients undergoing ERCP. To address this question, the goal of our study was to evaluate the risk of ERCP-related CM acute adverse reaction among patients known for iodine allergic-like reactions. Our hypothesis is that these reactions are very rare in this category of patients and may not justify prophylaxis.

## 2. Methods

This single centre retrospective study was approved by the University of Sherbrooke Research Ethics Board. Two reviewers analysed all the identified medical records for data extraction. All patients who underwent ERCP at our centre from January 2010 to December 2015 were identified for the study. Patients not exposed to iodine containing CM during ERCP were excluded. Collected data for all patients included demographics, reported allergic diathesis, reported iodine allergy, ERCP indication, and severe ERCP-related CM reaction. Severity of CM adverse reaction was defined as described in Table 1 [14]. Since it is our main study group, additional data were collected for patients known for iodine allergy: hospitalisation status, premedication prophylaxis, use of corticosteroid and antihistaminic, type of contrast media used, biliary manipulation during ERCP, post-ERCP duration of observation, and any ERCP-related CM adverse reaction. Patients who received corticosteroid with or without antihistaminic before ERCP were considered to have had premedication prophylaxis.

## 3. Results

From January 2010 to December 2015, 2295 ERCPs among 1766 patients were included in our study. Study population is described in Table 2. 828 ERCPs were performed in high risk of CM adverse reaction patients including 127 ERCPs among patients with prior adverse reaction to iodine containing product and 701 in patients reporting any other allergic diathesis. Results are described in Table 3. In our total study population, following 2295 ERCPs performed, no anaphylactoid or severe adverse reaction occurred. Only 1 (0.12%) ERCP-related CM moderate reaction was reported in a patient known for penicillin allergy. He had a delayed diffuse pruritic rash 24 hours following ERCP that was attributed to CM reaction after medical evaluation by the attending gastroenterology team and review of possible drug cause by the hospital pharmacist. This rash delayed the patient departure from the hospital but had a favorable response to medical treatment with IV corticosteroid and antihistaminic. 127 ERCPs were performed on 75 iodine allergic patients. Among these 75 high risk patients, prior reactions to CM were 10 (13.3%) severe reactions, 22 (29.3%) rashes, 16 (21.3%) oedema-angioedema reactions, and 27 (36.0%) others or unknown. CM used for ERCPs among iodine allergic patients were mostly low osmolality CM Omnipaque 240 (82.7%). Six (4.7%) ERCPs were done with prior premedication prophylaxis and 121 (95.3%) without. In both groups, no ERCP-related CM reaction occurred.

## 4. Discussion

As expected our study suggests that ERCP-related CM adverse reaction is very rare even among patients deemed to be at high risk. We found no severe adverse reaction after 2295 ERCPs performed including 127 ERCPs in patients with prior reaction to iodine product and 828 ERCPs in patients with any allergic diathesis. Only one moderate adverse reaction was reported. These results are similar to prior studies showing that ERCP-related CM reactions are very rare. Draganov and Forsmark prospectively studied 601 patients undergoing ERCP including 80 patients with a prior documented reactions to IV CM and 215 with other history of allergic reaction [15]. In their study, no adverse reactions associated with the administration of CM at the time of ERCP were observed in any of the patients. They concluded that prophylactic regimen against CM reaction before ERCP is unnecessary. Similarly, Moreira et al. reported performing ERCP in 16 patients with a previous history of minor reactions to radiological CM. None of them developed adverse reaction during ERCP or one hour after [16]. Catalano and Schwartz reported 25 patients with documented allergy to iodine undergoing ERCP [17]. No patients developed severe adverse reaction following CM injection during ERCP, but one patient known for a prior severe reaction to IV CM developed moderate nausea-vomiting and pruritus following ERCP even though he received premedication prophylaxis in the form of IV corticosteroid. Moreover, in a voluntary survey of more than 10,000 ERCP cases, Bilbao et al. reported no cases of serious adverse reaction to CM but 3 cases of erythema following CM injection during ERCP [1]. These results cannot be considered exact, however, due to the survey nature of the report.

Taken together these results suggest that minor or moderate ERCP-related CM reactions are possible but very rare. None of these studies reported a severe adverse reaction after CM injection during ERCP. The risk of a severe reaction would certainly argue for the use of premedication prophylaxis, and even though such reactions might be possible, we conclude on the basis of available clinical data that they must be exceedingly rare. Such a low risk certainly does not seem to justify premedication prophylaxis.

A cost-effectiveness study regarding CM premedication prophylaxis management strategies for ERCP is not available and will likely never exist since it seems unrealistic to prove any benefit with such rare and mostly benign adverse effect. It would still be valuable to know the costs of prophylaxis related to medication cost, ERCP procedures delays, and prophylaxis side effects as it could add arguments against premedication prophylaxis.

Our study main strength is the large cohort size. It is, to our knowledge, the largest cohort of CM reaction high risk patients undergoing ERCP. A large number of patients is necessary when looking for rare events. As such, we can conclude that among 828 patients with allergic diathesis, no serious adverse CM reaction occurred but 1 moderate CM reaction occurred. Also, among 127 patients with iodine product allergy undergoing ERCP no CM-related adverse event occurred. These results reinforce the safety of CM injection during ERCP even among high risk patients without premedication prophylaxis.

Our study has many limitations. The retrospective nature of the study made it impossible to have complete data and limited the characterization of allergic diathesis. It is likely that some patients had previous adverse reaction to IV CM contrast although they were not identified as such and it is likely that some were falsely identified as iodine allergic-like patients. Another limit of the study is the limited data on follow-up after ERCP for patients not known for iodine reaction. We chose to focus data collection on iodine allergic-like patients to achieve the study goal as well as possible. In this group, 52.7% had a follow-up time of 24 hours or less (and we presume that this proportion should be the same for the rest of the whole cohort). It is therefore possible that some delayed CM reactions were missed in data collection due to short follow-up time. Nevertheless, most delayed CM reactions are mild self-limited cutaneous reactions, and the vast majority of life-threatening reactions occur within minutes after CM injection. Severe adverse CM reaction would then certainly have not been missed. Finally, any monocentric study offers a limited external validity. Even though a retrospective study has inevitable flaws, it also allowed us to study a very large cohort which makes the strength of our study.

To conclude, our study results suggest that ERCP-related CM adverse reactions are very rare even among patients perceived at high risk for CM reaction. Therefore, we suggest that CM premedication prophylaxis for ERCP should not be given routinely neither in the general population, nor among patients with a past history of CM reaction.

## Figures and Tables

**Table 1:** Definition of CM adverse reaction severity.

Severity	Included reactions
Mild	Limited urticaria or pruritus Limited cutaneous oedema Nasal flushing Conjunctivitis

Moderate	Diffuse urticaria Facial oedema Diffuse cutaneous erythema Bronchospasm without hypoxia

Severe	Anaphylactoid shock Bronchospasm with hypoxia Laryngeal oedema with stridor or hypoxia Diffuse erythema with hypotension Diffuse or facial oedema with dyspnea

**Table 2:** Characteristic of study patients.

Characteristics	All patients (*N* = 1766)	Iodine allergic patients (*N* = 75)
Endoscopic procedure, no	2295	127
Age (years), mean ± SD	66.2 ± 28.1	69.6 ± 24.3
Gender		
(i) Male, no (%)	1067 (46.5)	46 (36.2)
(ii) Female, no (%)	1228 (53.5)	81 (63.8)
Year of the procedure		
(i) 2010, no (%)	345 (15.0)	-
(ii) 2011, no (%)	338 (14.7)	-
(iii) 2012, no (%)	349 (15.2)	-
(iv) 2013, no (%)	403 (17.6)	-
(v) 2014, no (%)	411 (17.9)	-
(vi) 2015, no (%)	449 (19.6)	-
Allergy		
(i) Yes, no (%)	828 (36.1)	127 (100.0)
(ii) No, no (%)	1467 (63.9)	0 (0.0)
Iodine Allergy		
(i) Yes, no (%)	127 (5.5)	127 (100.0)
(ii) No, no (%)	2168 (94.5)	0 (0.0)
History of severe iodine allergic reaction		
(i) Yes, no (%)	0 (0.0)	17 (13.3)
Prior iodine allergic reaction reported		
(i) Rash, no (%)	-	36 (28.3)
(ii) Oedema-angioedema, no (%)	-	34 (26.7)
(iii) Others-unknown, no (%)	-	57 (44.8)
ERCP indication		
(i) Lithiasis, no (%)	978 (42.6)	40 (31.5)
(ii) Cholangitis, no (%)	442 (19.3)	35 (27.5)
(iii) Main biliary obstructive lesion, no (%)	508 (22.1)	30 (23.6)
(iv) Stent manipulation, no (%)	228 (9.9)	14 (11.0)
(v) Chronic pancreatitis, no (%)	24 (1.0)	0 (0.0)
(vi) Biliary leak, no (%)	26 (1.1)	1 (0.8)
(vii) Other, no (%)	89 (3.9)	7 (5.5)
Hospitalised for ERCP		
(i) Yes, no (%)	-	77 (60.6)
(ii) No, no (%)	-	47 (37.0)
(iii) Unknown, no (%)	-	3 (2.4)

**Table 3:** Study results.

Results	All patients (*N* = 1766)	Iodine allergic patients (*N* = 75)
Endoscopic procedure included, no	2295	127
Post-ERCP anaphylaxis		
(i) Yes, no (%)	0 (0.0)	0 (0.0)
(ii) No, no (%)	2294 (99.9)	127 (100.0)
(iii) Not available, no (%)	1 (0.04)	-
Reported allergic reaction		
(i) Yes, no (%)	1 (0.0004)	0 (0.0)
(ii) No, no (%)	2294 (99.9)	127 (100.0)
Contrast induced adverse reaction		
(i) Rash, pruritus, no (%)	1 (0.0004)	0 (0.0)
(ii) Other, no (%)	0 (0.0)	0 (0.0)
ERCP details		
(i) Cholangiography, no (%)	-	124 (97.6)
(ii) Pancreatography, no (%)	-	66 (51.9)
(iii) Sphincterotomy, no (%)	-	67 (52.7)
(iv) Lithiasis extraction, no (%)	-	49 (38.5)
(v) Stent placement, no (%)	-	61 (48.0)
(vi) Biliary biopsy, no (%)	-	16 (12.6)
(vii) Brush cytology, no (%)	-	10 (7.8)
Contrast media		
(i) Omnipaque 240, no (%)	-	105 (82.7)
(ii) Visipaque 320, no (%)	-	19 (15.0)
(iii) Visipaque 270, no (%)	-	3 (2.4)
Observation period		
(i) 4 hours and less, no (%)	-	47 (37.0)
(ii) 4 to 24 hours, no (%)	-	20 (15.7)
(iii) More than a day, no (%)	-	60 (47.2)
